# In Vitro Cytotoxic Activity and Phytochemical Characterization (UPLC/T-TOF-MS/MS) of the Watermelon (*Citrullus lanatus*) Rind Extract

**DOI:** 10.3390/molecules27082480

**Published:** 2022-04-12

**Authors:** Heba A. El Gizawy, Alaadin E. El-Haddad, Yasmin M. Attia, Sally A. Fahim, Mai M. Zafer, Amr M. Saadeldeen

**Affiliations:** 1 Department of Pharmacognosy, Faculty of Pharmacy, October 6 University, Giza 12585, Egypt; hebaelgizawy@o6u.edu.eg (H.A.E.G.); alaa_elhaddad.ph@o6u.edu.eg (A.E.E.-H.); 2 Pharmacology Unit, Cancer Biology Department, National Cancer Institute, Cairo University, Kasr Al Eini Street, Fom El Khalig, Cairo 11796, Egypt; 3 Department of Biochemistry, School of Pharmacy, Newgiza University (NGU), Newgiza, km 22 Cairo-Alexandria Desert Road, Giza 12577, Egypt; sally.atef@ngu.edu.eg; 4 Microbiology and Immunology Department, Faculty of Pharmacy, Ahram Canadian University, Giza 12566, Egypt; maizafer@acu.edu.eg; 5 Department of Pharmacognosy, School of Pharmacy, Newgiza University (NGU), Newgiza, km 22 Cairo-Alexandria Desert Road, Giza 12577, Egypt; amr.saadeldeen@ngu.edu.eg

**Keywords:** *Citrullus lanatus*, cancer cell lines, apoptosis, VEGF, cell cycle

## Abstract

Reusing food waste is becoming popular in pharmaceutical industries. Watermelon (*Citrullus lanatus*) rind is commonly discarded as a major solid waste. Here, the in vitro cytotoxic potential of watermelon rind extracts was screened against a panel of human cancer cell lines. Cell cycle analysis was used to determine the induction of cell death, whereas annexin V-FITC binding, caspase-3, BAX, and BCL-2 mRNA expression levels were used to determine the degree of apoptosis. VEGF-promoting angiogenesis and cell migration were also evaluated. Moreover, the identification of phytoconstituents in the rind extract was achieved using UPLC/T-TOF-MS/MS, and a total of 45 bioactive compounds were detected, including phenolic acids, flavonoids aglycones, and their glycoside derivatives. The tested watermelon rind extracts suppressed cell proliferation in seven cancer cell lines in a concentration-dependent manner. The cytotoxicity of the rind aqueous extract (RAE) was higher compared with that of the other extracts. In addition to a substantial inhibitory effect on cell migration, the RAE triggered apoptosis in HCT116 and Hep2 cells by driving the accumulation of cells in the S phase and elevating the activity of caspase-3 and the BAX/BCL-2 ratio. Thus, a complete phytochemical and cytotoxic investigation of the *Citrullus lanatus* rind extract may identify its potential potency as an anticancer agent.

## 1. Introduction

Cancer is one of the leading causes of death globally [1], and the development of resistance of tumor cells to currently existing treatments urgently requires new anticancer medications [2]. Furthermore, several anticancer medications have life-threatening adverse effects because of their inability to distinguish between normal and tumoral cells [3,4]. Consequently, physicians and medical professionals are interested in identifying new anticancer compounds derived from natural sources, as well as from complementary herbal therapy [5,6,7].

Watermelon (*Citrullus lanatus*) is a major part of the Mediterranean diet, with the highest production of all the *Cucurbits* [8]. Watermelons are native to Africa, despite being a widely cultivated fruit with over 1000 varieties worldwide. Watermelon production in Ancient Egypt was documented by seeds found in Pharaonic tombs [9]. Watermelon is a natural diuretic that can be eaten fresh or processed as juice, jam, or seeds.

Watermelon rind is disposed of as a substantial solid waste and thereby contributes to the growing problem of solid food waste in the environment. Consequently, prospective methods for recovering bioactive compounds from *Citrullus lanatus* waste could have a significant impact on the agricultural food chain. Utilizing food wastes in diets and pharmaceuticals could enhance the food supply, promote health, and lessen the waste burden on the environment [10]. Furthermore, when compared to traditional waste processing techniques, such as composting or incineration, innovative strategies for the reuse and valorization of food waste are becoming increasingly popular in the food and pharmaceutical industries and are commonly referred to as “2nd generation food waste management” [11,12].

The rinds of various fruits have been used for several therapeutic purposes, including treatment for diarrhea, dysentery, acne, and wounds [13]. Watermelon rind can be classified as an agro-waste from which biologically and nutritionally valuable chemicals can be extracted and has also been used as a potential source of dietary fiber to improve health [14]. Watermelon rind has been shown to have free radical scavenging, antioxidant and antimicrobial activities owing to its phenolic compounds, such as 4-hydroxybenzoic acid, vanillin, quercetin, myricetin, and coumaric acid [7,15,16]. Citrulline is a nonessential amino acid that has considerable biological and nutritional importance and has been considered as a natural substitute for Viagra [17]. Citrulline is not commonly found in edible fruits, although it has been found in substantially higher concentrations in watermelon rind than in the pulp (24.7 and 16.7 mg/g, respectively) [18]. As a result, rind industrial wastes could be valuable as a natural supply of citrulline.

Importantly, only a few reports have previously described the composition of watermelon rind [19,20]. Therefore, this study aimed to identify the value of watermelon waste by using UPLC/T-TOF-MS/MS to characterize the chemical composition of rind aqueous extract (RAE). In addition, we also investigated the biological effects of RAE on the proliferation, cell cycle, apoptosis, and migration of human cancer cell lines: A549, Caco-2, H1299, HCT116, Hep2, HepG2, and MCF-7 in vitro compared to doxorubicin (DOX) as a reference cytotoxic standard.

## 2. Results and Discussion

### 2.1. Anticancer Activity Assays

#### 2.1.1. Cytotoxic Screening of Watermelon Rind Extracts

**Figure 1:**
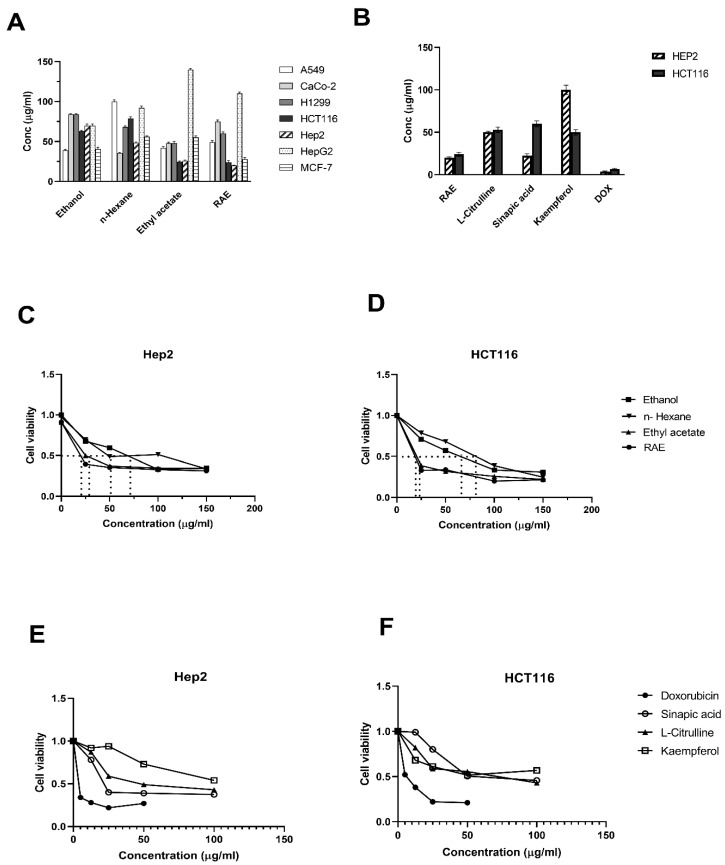
The effect of different concentrations of *Citrullus lanatus* extracts and standards on IC_50_ values (**A**–**D**) and cell viability (**E**–**F**) of various cancer cell lines. The cell viability was assessed after 48 h by SRB assay. The data are presented as the means and standard deviations of triplicate observations from three independent experiments.

The antiproliferation assay was used to assess the efficacy of the cell growth inhibitory effect of the watermelon rind extracts (ethanol, *n*-hexane, ethyl acetate, and aqueous) on 7 human cancer cell lines: A549, Caco-2, H1299, HCT116, Hep2, HepG2, and MCF-7 at 48 h after treatment. The comparative IC_50_ values are shown in Figure 1A,B. The most potent antiproliferative effect was induced by the rind aqueous extract (RAE) with IC_50_ values of 24 ± 2.4 and 20 ± 0.7 µg/mL in HCT116 and Hep2 cell lines, respectively. The RAE produced a dose-dependent decrease in cell viability in both Hep2 and HCT116 cells (Figure 1C,D). Moreover, the cytotoxic activity of the RAE was assessed in HCT116 and Hep2 cells compared with that of standard compounds such as kaempferol and sinapic acid, as well as L-citrulline and doxorubicin (DOX). These standards showed a dose-dependent decrease in cell viability in Hep2 and HCT116 cells (Figure 1E,F), with the most cytotoxic being DOX and sinapic acid in Hep2 cells with IC_50_ values of 3.5 ± 0.8 and 22.5 ± 2.1 µg/mL, respectively. Kaempferol had the least cytotoxic effect on Hep2 cells with an IC_50_ > 100 µg/mL. The most toxic compounds for HCT116 cells were DOX and kaempferol with IC_50_ values of 6.5 ± 1.1 and 50 ± 3.5 µg/mL, respectively. The RAE exhibited a more potent cytotoxic effect in HCT116 and Hep2 cells when compared with that of kaempferol, sinapic acid, or L-citrulline with IC_50_ values of 20 ± 1.2 and 24 ± 2.4 µg/mL, respectively.

These findings agreed with previous studies that reported a high proteasome inhibitor potential of *Citrullus lanatus* rind that can be useful for cancer treatment [21,22]. According to the US National Cancer Institute plant screening program, a crude plant extract has acceptable in vitro cytotoxic activity if the IC_50_ value is <20 µg/mL after incubating the cell lines for 48 and 72 h [23]. Therefore, we chose the RAE to further investigate the associated cell death processes, since this had the most potent cytotoxic effect on Hep2 and HCT116 cells.

#### 2.1.2. Annexin V-FITC/PI for Apoptosis Detection Using Flow Cytometry

**Figure 2:**
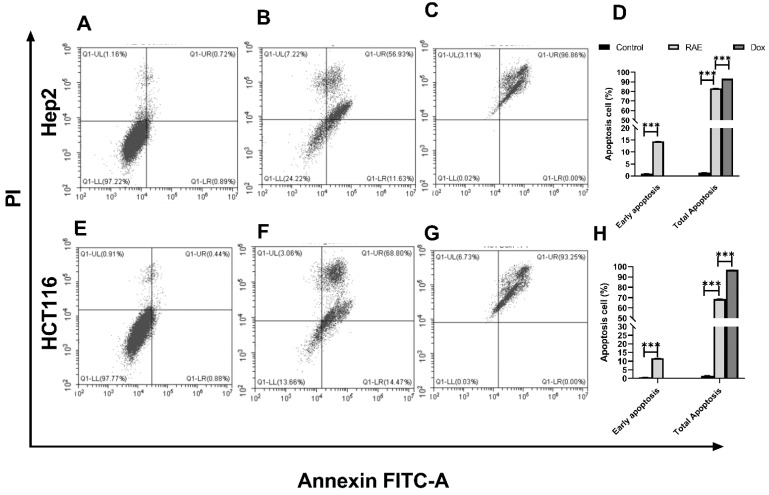
The effects of *Citrullus lanatus* RAE and DOX on apoptosis and necrosis in Hep2 and HCT116 cells. The control are untreated cells of Hep2 (**A**). Hep2 cells were treated with RAE at concentrations of 20 g/mL (**B**) and DOX at concentrations of 3.5 g/mL (**C**). The control are untreated HCT116 cells (**E**). HCT116 cells were treated with 42 g/ml of RAE (**F**) and 6.5 g/mL of DOX (**G**). Comparative analysis for early and total apoptosis for Hep2 (**D**) and HCT116 (**H**) cells. Flow cytometry was used to assess the induction of apoptosis/necrosis after 48 h. Cell numbers (percentages) from four different quadrants are represented in the representative dot plots (UL, Necrosis; UR, Late apoptosis; LL, live; LR, early apoptosis). *** Significant at *p* < 0.0001.

Analysis of the apoptosis and necrosis of human cancer cells was performed using flow cytometry to further investigate the mechanism of growth suppression on cancer cells. Phosphatidylserine is secreted on the extracellular surface by apoptotic cells and can be detected using Annexin V-labeled fluorescence, while necrotic cells can be detected by propidium iodide (PI) [24]. Hep2 and HCT116 cells were treated with RAE or DOX, and antibodies to Annexin V and PI staining were used to examine the results. The percentage of Hep2 and HCT116 cells that were found to be necrotic or apoptotic is shown in Figure 2. Total apoptosis was defined as the sum of early and late apoptosis percentages. The total apoptosis was significantly higher in RAE-treated cancer cell lines (Hep2: 82.47%, HCT116: 68.56%, Figure 2B,E) compared to the control group (Hep2: 1.32%, HCT116: 1.61%, Figure 2A,D). Treatment with DOX induced a total apoptosis of 93.25% in Hep2 (Figure 2C) and 96.86% in HCT116 (Figure 2F) cells. These results were consistent with the results from the SRB assay. A previous report indicated that the consumption of *Citrullus lanatus* could enhance apoptosis in colon cancer because of the presence of L-citrulline as a major constituent [25].

#### 2.1.3. Cell Cycle Analysis

**Figure 3:**
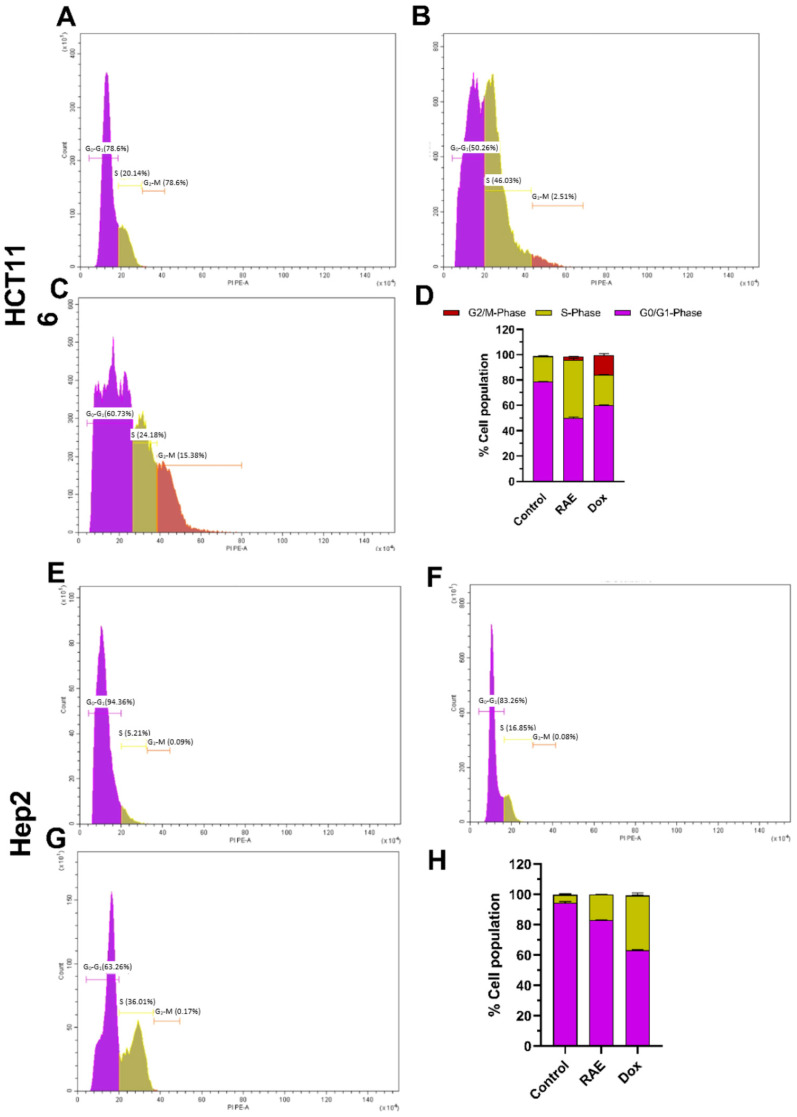
Cell cycle analysis by flow cytometry for Hep2 and HCT116 treated with IC_50_ of RAE and DOX for 48 h: **(A**,**E**) control, (**B**,**F**) RAE, and (**C**,**G**) DOX. (**D**,**H**) Comparative analysis for the sub-G_0_/G_1_, G_0_/G_1_, S, and G_2_/M phases across different groups. All results are expressed as percentages of the cell population with mean ± SD of three experiments.

The effect of the watermelon rind extracts on the cell cycle distribution of Hep2 and HCT116 cells was evaluated after 48 h to ensure whether an antiproliferative effect induced by RAE was related to cell cycle arrest. When treated with either RAE or DOX, the distribution of distinct phases changed dramatically (Figure 3). The proportion of treated cells in the S and G_2_/M stages was significantly higher than that in the control group. The cell cycle analysis using flow cytometry in HCT116 cells revealed that the RAE and DOX treatments both triggered cell cycle arrest at the S (46.03%) and G_2_/M (15.38%) phases, respectively (Figure 3A–D). However, the RAE and DOX treatments resulted in an increase in the number of Hep-2 cells arrested at the S phase (16.65% and 36.01%, respectively) (Figure 3E–H). Plant extracts are known to cause cytotoxicity through a variety of mechanisms, including cell cycle arrest and apoptosis. The potential of anticancer drugs to induce cell cycle arrest in cancer cells has been commonly used to assess their potential [26]. The tumor suppressor gene p53 has been reported to restrict cell proliferation by cell death and arresting of the cell cycle in the G_1_/S phase [27].

#### 2.1.4. Wound Healing Assay

**Figure 4:**
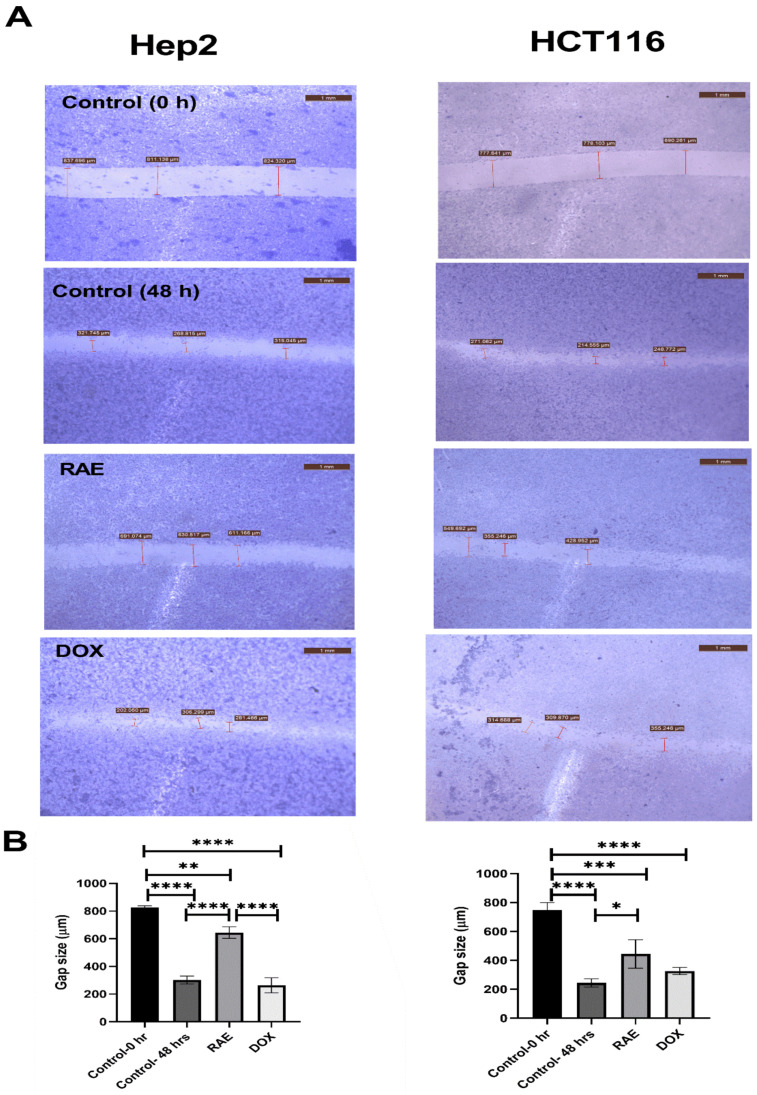
Effect of RAE and DOX on cell migration in Hep2 and HCT116 cells (**A**). Scratching was done with a 10-µL pipette tip. Quantitative representation of the migration of Hep2 and HCT116 by the wound healing assay (**B**). The data was presented as the mean and standard deviation. The one-way ANOVA test was used to examine statistical differences. * Significant at *p* < 0.05, ** significant at *p* < 0.001, *** significant at *p* < 0.0001, and **** significant at *p* < 0.00001.

The effects of RAE and DOX on the progression and migration of Hep2 and HCT116 cells were evaluated for 48 h. The wound in the control group was healed 48 h after scratching a monolayer of cells. When cells were treated with RAE IC_50_, the wound healing of the scratched area was significantly delayed compared with that in both the untreated cells and DOX-treated cells (Figure 4A). The RAE significantly decreased the wound closure by 53.14% (*p* < 0.00001) in Hep2 and 44.9% (*p* < 0.05) in HCT116 cells compared with that of the control group. DOX treatment did not affect the cell migration in either cell line when compared with that in the control group after 48 h. However, the RAE decreased closure by 59.13% (*p* < 0.00001) when compared with that in DOX-treated Hep2 cells only (Figure 4B).

Cell migration in vitro is an indication to cancer metastasis, which is the leading cause of cancer death worldwide [28,29]. In our study, the RAE significantly inhibited cell migration and metastatic progression in Hep2 and HCT116 cells and may therefore represent a potent anticancer agent in decreasing cancer metastasis.

#### 2.1.5. VEGF, Caspase-3, BAX, and BCL-2 mRNA Expression Levels

**Figure 5:**
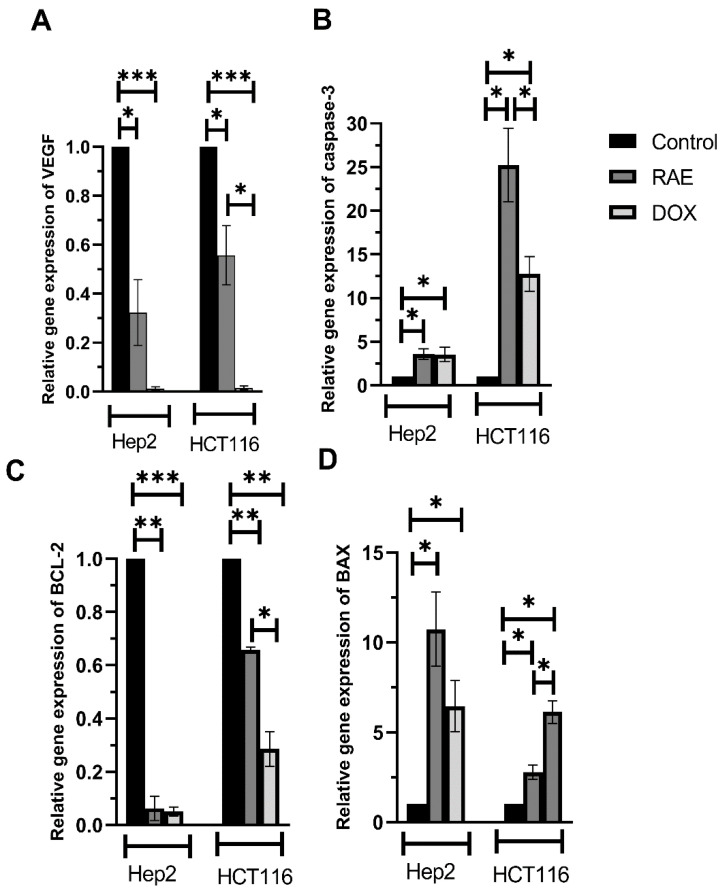
Gene expression level of VEGF (**A**) and apoptosis-regulating proteins like BAX (**B**), BCL-2 (**C**), and caspase-3 (**D**) in Hep2 and HCT116 cells treated with the RAE or DOX standard at 48 h. Values were expressed as the mean ± SD. Statistical differences were analyzed with the one-way ANOVA test. * Significant at *p* < 0.05, ** significant at *p* < 0.001, and *** significant at *p* < 0.0001.

The expression of angiogenic VEGF mRNA was evaluated to determine the molecular gene expression patterns for wound healing-related genes. As shown in Figure 5A, treatment with RAE significantly suppressed the expression of VEGF mRNA by 68% (Hep2) and 44% (HCT116) when compared to the control group. However, VEGF mRNA expression was suppressed more effectively in HCT116 cells by DOX treatment when compared with that induced by the RAE treatment (*p* < 0.05).

To understand the mechanism of apoptosis induction in Hep2 and HCT116 cells, the mRNA expression level of apoptosis-regulating proteins such as BAX (Figure 5B), BCL-2 (Figure 5C), and caspase-3 (Figure 5D) were assessed. Treatment with RAE significantly suppressed the mRNA expression level of the antiapoptotic protein BCL-2 by 93.8% (Hep2) and 35% (HCT116) when compared with that in the control group. DOX-treated cells showed a significant decrease in BCL-2 mRNA expression by 56.9% when compared with that in HCT116 cells following the RAE treatment. Moreover, the treatment with RAE increased expression of the proapoptotic protein BAX (Hep2: 10.7-fold, HCT116: 2.8-fold) and caspase-3 (Hep2: 3.6-fold, HCT116: 25.2-fold) when compared with that in the control cells. Furthermore, DOX-treated HCT116 cells showed an increase in BAX mRNA levels when compared with those in the RAE-treated HCT116 cells, whereas the RAE treatment increased the caspase-3 levels when compared with those in DOX-treated Hep2 cells.

Antiapoptotic and the proapoptotic proteins play a significant role in the regulation of apoptosis induction by cells [30]. In this study, apoptosis was induced in Hep2 and HCT116 cells, because the treatment with RAE elevated the expression of proapoptotic proteins BAX and caspase-3 while decreasing that of the antiapoptotic protein BCL-2. Moreover, VEGF and the associated receptor (VEGFR) are known to play a major role in pathological angiogenesis, such as that occurring in cancer [31]. Previous studies have also shown that the RAE from *Citrullus lanatus* prevented prostatic hyperplasia, as characterized by an increase in the levels of VEGF [32,33].

### 2.2. Antimicrobial Activity Assays

The sensitivity of 3 standard microbial strains to the extracts was assessed using the agar well-diffusion method. The standard bacterial strains were sensitive to doxycycline, while *C. albicans* was sensitive to amphotericin B. However, the watermelon rind extracts did not affect the growth of any of the test organisms.

### 2.3. Chemical Characterization of Watermelon Rind Extract Using UPLC/T-TOF-MS/MS

Our biological investigations indicated that RAE was the most biologically active one; thus, its chemical quantification and characterization were performed. Phenolics may be the principal hydrophilic antioxidant compounds in watermelon. Total phenolic content (TPC) was present at 178.84 ± 0.64-mg gallic acid equivalent/g, while total flavonoid content (TFC) was present at 80.12 ± 1.41-mg rutin equivalent/g in the RAE. This is comparable to the levels reported previously [34].

**Figure 6:**
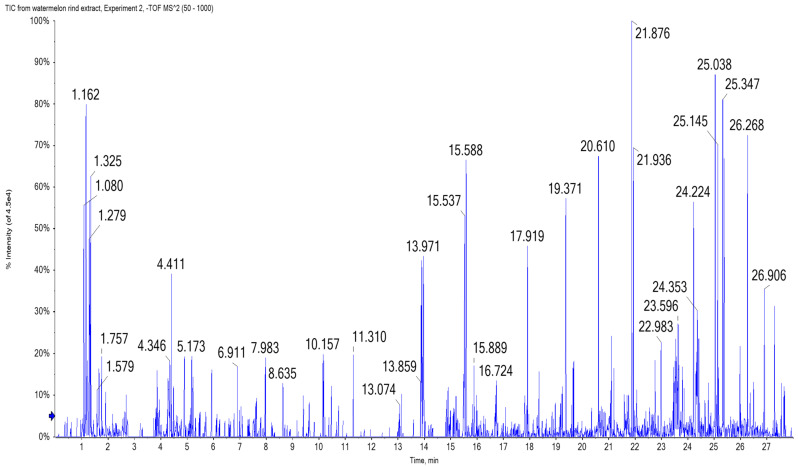
UPLC/T-TOF-MS/MS chromatogram of the watermelon (*Citrullus lanatus*) rind aqueous extract in negative ionization mode.

**Figure 7:**
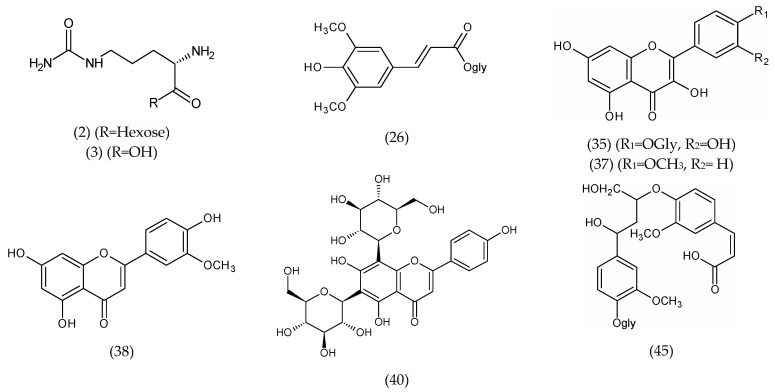
Representative structures of various metabolites (flavonoids, phenolics, and amino acids) identified in the rind aqueous extract of *Citrullus lanatus*.

The identification of phytoconstituents using LC-MS/MS spectrometry is an efficient method for the screening and identification of phytoconstituents in plants. This is the first study to use UPLC/T-TOF-MS/MS to characterize the metabolites in watermelon rind. Metabolite assignments were made by comparing retention times and MS data (accurate mass and fragmentation pattern) with those described in the literature for *Citrullus* species, as well as accessing online public databases. A total of 45 phytochemical substances have been provisionally identified, the majority of which are phenolic acids (hydroxybenzoic and sinapic acids), flavonoids aglycones, and their glycosides derivatives, all of which reflect plants’ various biological activities [35] (Table 1 and Figure 6 and Figure 7).

- *Hydroxybenzoic acid derivatives*

The proposed method has been useful for the characterization of 4 hydroxybenzoic acid derivatives. The parent ion observed at *m/z* 301.1045 has been suggested to be phloroglucinol-*O*-glycuronic acid (20). The ion fragment detected at *m/z* 125.0360 may have been produced by the loss of glucuronic acid [M-H-glycuronic acid]^−^; moreover, the ion peak at *m/z* 175.0781 indicates the loss of phloroglucinol [M-H-phloroglucinol]^−^ [36]. Salicylic acid (23) showed a deprotonated molecule [M-H]^−^ at *m/z* 137.0237 and an ion fragment detected at *m/z* 93.0346 [M-H-CO_2_]^−^, which have been already identified in Cucurbitaceae [37].

- *Hydroxycinnamic acid derivatives*

*O*-feruloylquinide (24) was indicated by the deprotonated molecule [M-H]^−^ at *m/z* 349.0899 and a daughter ion at *m/z* 193.0502 similar to that for ferulic acid; this compound has been described previously in Cucurbitaceae [38]. Caffeoyl hexoside (25) shows a deprotonated molecule [M-H]^−^ at *m/z* 341.0731, and the neutral loss of 162 amu of hexose moiety was observed in MS2 fragmentation at *m/z* 179.0557 [M-H-hexose]^−^ [37]. The data obtained from the T-TOF-MS analysis showed a deprotonated molecule [M-H]^−^ at *m/z* 385.1158 and a daughter ion at *m/z* 223.0546 [37], which corresponded to a loss of a hexose moiety that was proposed as sinapic acid hexoside (26). The spectrum showed two deprotonated molecular ions at *m/z* 471.1469 (28 and 29) and an ion fragment at *m/z* 193.0516 [M-H-sugar]^−^; this latter ion may have originated from the neutral loss of pentose and deoxyhexose moieties (−278 Da). Depending on the data obtained from the literature [39], compounds 28 and 29 were tentatively identified as *O*-feruloyl-pentosyl-deoxyhexose. Decaffeoyl acetoside or descaffeoyl verbascoside (30) showed a deprotonated molecule [M-H]^−^ at *m/z* 461.1656 and daughter ions at *m/z* 309.1171 [M-H-hydroxytyrose]^−^ and 147.0672 [M-H-hydroxytyrose-hexose]^−^ [36]. Ferulic and chlorogenic acids (31 and 32) were also detected [40].

- *Flavonoid derivatives*

Baicalein-*O*-glycuronide (34) showed a molecular ion [M-H]^−^ at *m/z* 445.0681. Three isomers at 15.67, 16.75, and 20.33 min (37–39) showed a molecular peak [M-H]^−^ at *m/z* 299.0564 and were assigned a molecular formula (C_16_H_11_O_6_) tentatively characterized as trihydroxy-methoxyflavone (this may include kaempferide or chrysoeriol) [36]. Two peaks were detected at retention times 21.02 and 21.11 min at *m/z* 593.1573 (C_27_H_29_O_15_). The precursor ion has already been mentioned in the bibliography in the Cucurbitaceae family [37,41] as apigenin-6,8-C-di-hexoside or vicenin-2 (40 and 41). Kaempferol-7-neohesperidoside (42) showed a deprotonated molecule [M-H]^−^ at *m/z* 593.1442. Flavonol derivatives also identified as quercetin hexoside (35) showed a molecular ion [M-H]^−^ at *m/z* 463.0403; the neutral loss of 162 amu of a hexose moiety was observed in MS2 fragmentations at *m/z* 301.1014 [M-H-hexose]^−^. Quercetin rhamnoside (quercitrin) (36) was detected at 7.50 min with a molecular ion at *m/z* 447.0911 and a daughter ion at *m/z* 301.0372 [M-H-rhamnose]^−^, which corresponds to the presence of quercetin, achieved by the loss of the rhamnose moiety [M-H-146]^−^ [37].

- *Amino and organic acid derivatives*

Amino and organic acids were abundant in the RAE and eluted early in the chromatogram. Among these acids, citrulline (3) was identified by a peak at *m/z* 174.0874 [M-H]^−^ and its hexoside derivative (2) at *m/z* 336.1411 [M-H]^−^, and the neutral loss of 162 amu of the hexose moiety was observed in MS2 fragmentations at *m/z* 174.0882 [M-H-hexose]^−^ in comparison with published data [18] as being the major amino acids. Citrulline is reported to be an efficient hydroxyl radical scavenger and to have a strong antioxidant capacity [42]. Here, the identified amino acids also included oxoproline and phenylalanine. The common neutral loss of 44 Da was observed in MS2 fragmentations of organic acids due to the loss of CO_2_ as in tartaric (9), shikimic, maleic, citramalic, and citraconic acids (10–13) [43]. The identified organic acids also include citric and malic acids (7 and 8).

- *Miscellaneous metabolites*

A tentatively identified coumarin, esculetin hexoside (43), was detected at *m/z* 339.0974 [M-H]^−^. Leachianol G (44), an oligostilbens resveratrol dimer, showed a deprotonated molecule [M-H]^−^ at *m/z* 471.1469 [36]; however, glehlinoside C (45), a neolignane glycoside, showed a molecular ion [M-H]^−^ at *m/z* 551.1824 [36].

**Table 1:** Tentatively identified metabolites via UPLC/T-TOF-MS/MS from watermelon (*Citrullus lanatus*) rind aqueous extract using negative ionization mode.

Proposed Compounds		Formula	Rt	[M-H]^−^ *m/z*	Ref. Mass	Diff. (ppm)	Ms^2^ (Characteristic Fragments)	Ref.
**Amino acid derivatives**								
1	Oxoproline	C_5_H_6_NO_3_	1.19	128.0341	128.0342	−0.8	84.0190 [M-H-CO_2_]^−^	--
2	Citrulline-*O*-hexoside	C_12_H_22_N_3_O_8_	1.23	336.1411	336.1401	2.8	174.0882 [M-H-hexose]^−^, 131.0823	--
3	Citrulline	C_6_H_12_N_3_O_3_	1.28	174.0874	174.0873	0.8	131.0822, 113.0711, 70.0651	[18]
4	3,4-Dihydroxy-L-phenylalanine (DOPA)	C_9_H_11_NO_4_	1.45	196.0714	196.0615	2.9	160.8441, 151.0531 [M-H-COOH]^−^, 67.03075	--
5	Arginine	C_6_H_13_N_4_O_2_	1.72	173.1049	173.1033	9.2	131.0827, 89.0190	[44]
6	Phenylalanine	C_9_H_10_NO_2_	2.28	164.0704	164.0706	−1.3	147.0470 [M-H-OH]^−^, 119.0494 [M-H-COOH]^−^, 103.0607	[43]
**Organic acid derivatives**								
7	Citric acid / Isocitrate	C_6_H_7_O_7_	1.08	191.0195	191.0186	4.3	173.0114 [M-H-H_2_O]^−^, 129.0236 [M-H-H_2_O-CO_2_]^−^, 111.0089	[43]
8	Malic acid	C_4_H_5_O_5_	1.08	133.0136	133.0132	3.3	115.0030 [M-H-H_2_O]^−^, 71.0140 [M-H-H_2_O-CO_2_]^−^	[43]
9	Tartaric acid	C_4_H_5_O_6_	1.13	149.0092	149.0081	7.3	105.0021 [M-H-CO_2_]^−^	--
10	Shikimic acid	C_7_H_10_O_5_	1.17	173.0115	173.0455	−11.8	155.0439, 136.9845, 129.0206 [M-H-CO_2_]^−^	
11	Maleic acid	C_4_H_3_O_4_	1.19	115.0015	115.0026	−9.2	71.0121 [M-H-CO_2_]^−^	--
12	Citramalic acid / Citramalate	C_5_H_7_O_5_	1.33	147.0291	147.0288	2.0	129.0176 [M-H-H_2_O]^−^, 103.0401 [M-H-CO_2_]^−^, 87.0087, 61.9883	--
13	Citraconic acid	C_5_H_6_O_4_	1.70	128.9589	129.0193	2.1	101.0132, 84.9877 [M-H-CO_2_]^−^, 55.0225	--
**Sugar derivatives**								
14	Mucate (Galactarate)	C_6_H_9_O_8_	1.05	209.0289	209.0292	−1.4	191.0227 [M-H-H_2_O]^−^, 165.3369 [M-H-CO_2_]^−^, 147.0334 [M-H-H_2_O-CO_2_]^−^, 133.0136	--
15	Gluconic acid	C_6_H_11_O_7_	1.14	195.0503	195.0499	2.0	129.0198, 75.0085	--
16	Tagatose	C_6_H_11_O_6_	1.37	179.0553	179.0550	1.3	89.0244, 71.0155, 59.0144	--
17	Trehalose	C_12_H_22_O_11_	1.44	341.1078	341.1089	0.4	305.0897, 179.0544, 163.0495, 129.0205	--
18	Ribitol (xylitol)	C_5_H_11_O_5_	1.53	151.0607	151.0601	3.7	101.0217, 89.0241, 71.0148	--
19	Iditol	C_6_H_13_O_6_	1.53	181.0725	181.0707	10.1	163.0557 [M-H-H_2_O]^−^, 101.0251, 96.9682	--
**Hydroxybenzoic acid derivatives**								
20	Phloroglucinol glycuronide	C_12_H_13_O_9_	1.23	301.1045	301.0565	1.2	175.0781 [M-H-phloroglucinol]^−^, 125.0360 [M-H-glycuronic acid]^−^	[36]
21	Salicin benzoate	C_20_H_21_O_8_	1.41	389.1216	389.1231	−3.8	343.1081	[36]
22	3-Phenyllactic acid	C_9_H_10_O_3_	1.23	165.0391	165.0557	5.1	147.0340, 129.0155, 89.0291, 72.9876	--
23	Salicylic acid	C_7_H_5_O_3_	3.30	137.0237	137.0233	2.8	93.0346 [M-H-CO_2_]^−^	[37]
**Hydroxycinnamic acid derivatives**								
24	*O*-Feruloylquinide	C_17_H_17_O_8_	1.28	349.0899	349.0918	−5.5	193.0502 [ferulic acid]^−^	[38]
25	Caffoyl hexoside	C_15_H_18_O_9_	1.23	341.0731	341.0878	−1.1	179.0557 [M-H-hexose]^−^	[38] [37]
26	Sinapic acid hexoside (isomer 1)	C_17_H_21_O_10_	1.86	385.1158	385.1129	7.4	223.0546 [M-H-hexose]^−^	[37]
27	Sinapic acid hexoside (isomer 2)	C_17_H_21_O_10_	1.97	385.1339	385.1129	5.0	223.0631 [M-H-hexose]^−^	[37]
28	O-feruloyl-pentosyl-deoxyhexose (isomer 1)	C_21_H_27_O_12_	4.65	471.1469	471.1497	6.7	193.0516 [M-H-sugar]^−^	[39]
29	O-feruloyl- pentosyl-deoxyhexose (isomer 2)	C_21_H_27_O_12_	4.78	471.1467	471.1497	−6.0	193.0555 [M-H-sugar]^−^	[39]
30	Decaffeoyl acetoside/ Descaffeoyl verbascoside	C_20_H_29_O_12_	5.47	461.1656	461.1654	0.5	309.1171 [M-H-hydroxytyrose]^−^, 147.0672 [M-H-hydroxytyrose- hexose]^−^	[36]
31	Ferulic acid	C_10_H_9_O_4_	8.91	193.0499	193.0495	2.0	178.0285, 134.0360	[40]
32	Chlorogenic acid	C_16_H_18_O_9_	15.55	353.1937	353.0878	5.6	353.1898, 352.0573	--
33	*p*-Coumaric acid hexoside	C_15_H_18_O_8_	15.89	325.1853	325.1753	−5.4	325.1642, 325.1770	--
**Flavonoid derivatives**								
34	Baicalein-*O*-glycuronide	C_21_H_18_O_11_	1.42	445.0681	445.0776	3.5	445.1562	--
35	Quercetin hexoside	C_21_H_20_O_12_	1.47	463.0403	463.0882	0.1	301.1014 [M-H-hexose]^−^, 61.9916	--
36	Quercetin rhamnoside (Quercitrin)	C_21_H_19_O_11_	7.50	447.0911	447.0922	−2.5	301.0372 [M-H-rhamnose]^−^, 174.9577	[37]
37	Trihydroxy-methoxyflavone Kaempferide (isomer 1)	C_16_H_11_O_6_	15.67	299.0511	299.0550	8.2	284.0304	--
38	Trihydroxy-methoxyflavone (Chrysoeriol) (isomer 2)	C_16_H_11_O_6_	16.75	299.0564	299.0550	4.7	271.0251	[36]
39	Trihydroxy-methoxyflavone (isomer 3)	C_16_H_11_O_6_	20.33	299.0547	299.0550	−1.0	284.0340, 271.0251	[36]
40	Apigenin-6,8-C-di-hexoside (Vicenin-2) (isomer 1)	C_27_H_29_O_15_	21.02	593.1573	593.1501	7.0	--	[37]
41	Apigenin-6,8-C-di-hexoside (Vicenin-2) (isomer 1)	C_27_H_29_O_15_	21.11	593.1505	593.1501	0.7	--	[37]
42	Kaempferol-7-neohesperidoside	C_27_H_29_O_15_	21.12	593.1442	593.1501	−5.9	--	--
**Miscellaneous metabolites**								
43	Esculetin hexoside	C_15_H_16_O_9_	1.36	339.0974	339.0721	−2.2	303.1098, 296.0112, 213.0935, 134.0744	--
44	Leachianol G	C_28_H_23_O_7_	4.78	471.1469	471.1438	6.4	355.1081, 193.0555	[36]
45	Glehlinoside C	C_26_H_31_O_13_	22.62	551.1824	551.1759	1.7	--	[36]

### 2.4. Docking of Citrulline on Caspase-3 and VEGF Kinase Proteins

**Figure 8:**
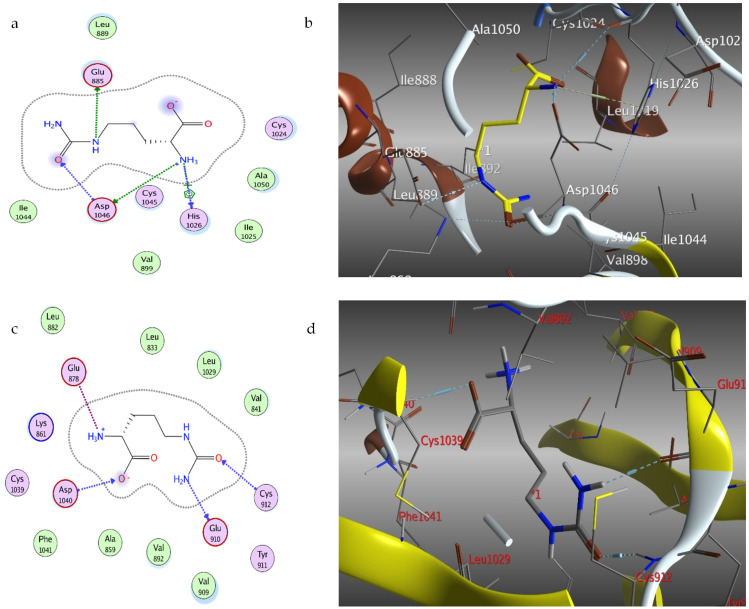
Citrulline (yellow)-binding modes, 2D diagram (**a**) and 3D representation (**b**) with 2P2I and 2D diagram (**c**) and 3D representation (**d**) with 3HNG VEGF kinase-binding pocket.

The molecular docking of citrulline was performed for the first time on caspase-3 and VEGF kinase proteins (Table 2). Citrulline was fitted on enzymes and displayed moderately to promising binding affinities with VEGF proteins ranging from −4.19 to −5.09 kcal mol^−1^. The most hopeful in silico activity was recorded on 2P2I with a binding affinity −4.79 kcal mol^−1^ and interact by four H-bonds with HIS1026, ASP1046, and GLU885 (Figure 8). Citrulline was fitted on caspase-3 with binding affinities of −4.24 kcal mol^−1^ and interacts by four H-bonds between the amino and carboxylic groups and MET61, GLY122, and ARG207. Moreover, it also interacts with four ionic bonds with ARG207 (Figure 8).

## 3. Materials and Methods

### 3.1. Chemicals

Gallic acid, rutin, citrulline, sinapic acid, kaempferol, and solvents (HPLC grade) were purchased from Merk (Zug, Switzerland). Dimethylsulfoxide (DMSO) and other reagents were of high analytical grade.

### 3.2. Plant Material and Extraction Process

Watermelon (*Citrullus lanatus* Thunb., cultivars: Giza (Egyptian cv)) fruits were grown in a garden under normal conditions in Egypt between June and July 2019. Fruits were washed with tap water, and the rind was separated and dried. Dried rind (1 kg) was powdered and extracted in a Soxhlet (Alderich^®^ soxhlet, Darmstadt, Germany) with ethanol (4 × 500 mL, 80%). The dried ethanol extract was fractionated between *n*-hexane, ethyl acetate, and water. Extracts were filtered and then evaporated using a Rotavapor^®^ (BÜCHI, Flawil, Switzerland) [45]. The obtained dried extracts were used for biological and chemical investigations.

### 3.3. Experimental Design

The in vitro cytotoxic potential of watermelon rind extracts was screened against a panel of human cancer cell lines. Various anticancer assays were performed. Cell cycle analysis was used to determine the induction of cell death, whereas annexin V-FITC binding, caspase-3, BAX, and BCL-2 mRNA expression levels were used to determine the degree of apoptosis. VEGF-promoting angiogenesis and cell migration were also evaluated. In addition, the antimicrobial effect was examined. Moreover, the identification of phytoconstituents in the biologically active RAE was achieved using UPLC/T-TOF-MS/MS.

### 3.4. Anticancer Activity

#### 3.4.1. Cell Lines

All cancer cell lines: A549 (lung), Caco-2 (colon), H1299 (non-small cell lung), HCT116 (colorectal), Hep2 (larynx), HepG2 (liver), and MCF-7 (breast) used in this investigation were supplied by the American Type Culture Collection (ATCC, Manassas, VA, USA). Serial subculturing was used to maintain tumor cells at the National Cancer Institute in Cairo, Egypt. All the human tumor cell lines were cultured and maintained in RPMI-1640 medium supplemented with 1% penicillin/streptomycin and 10% fetal bovine serum (FBS). Cells were subcultured to pre-confluence and incubated at 37 °C in a humidified environment with 5% CO_2_.

#### 3.4.2. Cytotoxicity Assay

The cytotoxicity of rind extracts (ethanol, *n*-hexane, ethyl acetate, and RAE) against 7 cancer cell lines was investigated. Human cancer cell lines were seeded at 2 × 10^3^ cells/well in 96-well microtiter plates in supplemented RPMI-1640 media and FBS and then grown for 24 h. Cells were treated for 48 h with the serial dilution of each extract (0–150 μg/mL) or the standards (0–100 µg/mL). The remaining surviving cells were tested for cytotoxicity using the sulphorhodamine-B (SRB) assay [46]. The optical density (OD) of each well was measured spectrophotometrically at 570 nm using an ELISA microplate reader.

The surviving fraction equals the OD of treated cells divided by that of the control cells (cells that were not exposed to the extract). All experiments were performed in triplicate, and the results were expressed as the mean ± SD. The 50 percent inhibitory concentration (IC_50_) was calculated from dose–response curves using GraphPad Prism 8.4.2. For further analysis, the experiment with the lowest IC_50_ was chosen, and HCT116 and Hep2 cells that had been exposed to RAE were therefore chosen for the next experiments.

#### 3.4.3. Wound Healing Assay

The wound healing assay was used to assess the inhibition of cell migration and metastases. HCT116 and Hep2 cells were seeded into 6-well plates and allowed to develop to 80–90% confluency before being scratched/wounded uniformly in each well with a 10-μL pipette tip. To eliminate debris, the cells were washed in sterile PBS before being treated with either the RAE or DOX standard. Wound closure was observed immediately (0 h) and after 48 h with an inverted microscope (DFC290, Leica, Wetzlar, Germany). All experiments were repeated three times [47].

#### 3.4.4. Annexin V-FITC/PI for Apoptosis Detection

For gene expression and flow cytometer assays, the Hep2 and HCT116 cells were grown at a concentration of 1 × 10^6^ cells/mL in 75-cm^2^ Falcon flasks in RPMI-1640 media supplemented with 10% FBS and antibiotics for 24 h. Following removal and refreshment with a new medium, incubation was continued for 48 h in the presence of RAE or DOX in accordance with the IC_50_ values obtained from the SRB assay for each cell line. Treatment-free cells were used as negative controls. Trypsin EDTA was used to extract cells.

A FITC Annexin V kit was used to further investigate apoptosis. All aspirates were centrifuged for 5 min at 13,000 rpm at 4 °C before being rinsed with cold PBS. The cells were extracted and centrifuged for 5 min at 2500 rpm at 10 °C. The supernatant was discarded, and the pellet was rinsed with 1-mL PBS before being centrifuged at 2500 rpm for 5 min at 10 °C. Pellets were incubated for 30 min in PBS (50 μL) with Annexin V-FITC and PI. After incubation, a flow cytometry analysis was performed with 300 μL of PBS-containing incubated cells into the flow cytometer.

#### 3.4.5. Cell Cycle Analysis

Flow cytometry was used to examine the cell cycle. Cells were collected by trypsinization and fixed in 70% (*v/v*) ethanol at 4 °C overnight after treatment. Fixed cells were resuspended in PBS containing 50-µg/mL PI and 0.1-mg/mL RNaseA (Beyotime, Jiangsu, China) after being washed twice with PBS. Cells were incubated for 30 min at 37 °C in the dark and then assessed using a LSR II FACS flow cytometer (BD Biosciences, Franklin Lakes, NJ, USA) to determine how many cells were in the G_1_, S, and G_2_/M phases of the cell cycle.

#### 3.4.6. VEGF, BAX, BCL-2, and Caspase-3 mRNA Expression Levels

A total RNA purification kit (Jena Bioscience, Munich, Germany) was used to extract the total RNA from treated and control samples. The cDNA archive kit (Applied Biosystems, Foster City, CA, USA) was used to convert RNA to cDNA. qPCR reactions contained 1 μL of cDNA, 25 μL of GoTaq PCR master mix (Promega Co., Madison, WI, USA), 0.25 μL of CXR reference dye, 1 μL of forward and reverse primers (Table 3), and DNase-free water to 50 μL. qPCR was performed using a 7500 Real-Time PCR System (Applied Biosystems, Foster City, CA, USA), and all analyses were done in triplicate. Results were reported as relative expression levels after being normalized to GAPDH using the 2^−ΔΔCt^ approach [48]. Primer gene sequences were taken from the NCBI database. Primers were designed and their specificities were checked using Primer3Plus and Blast, respectively [49].

### 3.5. Antimicrobial Activity

#### 3.5.1. Agar Well-Diffusion Assay

The antibacterial activity of the extracts was tested against *Staphylococcus aureus* (ATCC 6358) and *Escherichia coli* (ATCC 25923) using the agar well-diffusion method. Extracts (500 mg/mL in DMSO) were separately sterilized by filtration using 0.22-µm membrane filters (Indiamart, Vadodara, India). The surface of the Muller–Hinton agar plate was infected by spreading a volume of microbial inoculum across the entire agar surface. Then, a sterile cork borer was used to aseptically punch a hole with a diameter of 6–8 mm, and each extract (100 µL) was added to individual wells. Agar plates were incubated under the appropriate conditions for the test microorganism [50]. Standard doxycycline and amikacin antibiotics were used as controls. DMSO was used as a negative control that showed no bacterial inhibition.

#### 3.5.2. Antifungal Activity

The agar well-diffusion method was used to investigate the antifungal activity of the extracts [51]. The extract activity was compared with that of *Candida albicans* ATCC 10231, which was spectrophotometrically adjusted to a final concentration of 0.5 McFarland standard at 530 nm. The inhibition zones were assessed after 24 h of incubation at 28 °C. Amphotericin, a standard antibiotic, was employed as a control. Each experiment was performed three times with the mean values reported.

### 3.6. Total Phenolic and Flavonoid Contents

A stock solution (1 mg/mL in methanol) and serial dilutions of gallic acid and rutin (as standards for phenolic and flavonoid compounds, respectively) were prepared. The RAE was prepared at 10 mg/mL in DMSO. The Folin–Ciocalteu [35,52] and aluminum chloride techniques [53] were used to determine TPC and TFC, respectively. Absorbances were measured at 630 and 420 nm, respectively, using a microplate reader (FluoStar Omega, BMG LABTECH, Ortenberg, Germany). Data were represented as the means ± SD.

### 3.7. UPLC/T-TOF-MS/MS Analysis

The RAE was analyzed using UPLC/T-TOF-MS/MS in negative mode [54] with an ExionLC Triple TOF 5600+ system (SCIEX, Framingham, MA, USA) operated at 40 °C and equipped with an X select HSS T3 C-18 column (Waters Corporation, Milford, CT, USA, 2.5 μm, 2.1 × 150 mm). The RAE (50 mg) was dissolved in the solvent working solution (deionized water:methanol:acetonitrile at 50:25:25), sonicated (10 min), and then centrifuged (10,000 rpm, 10 min). The extract (1 µg/µL, 10 µL) was injected using the mobile phase, where solvent A was ammonium formate buffer (5 mM, pH 8) containing methanol (1%), and solvent B was acetonitrile (100%). A gradient elution at a flow rate of 0.3 mL/min was used as follows: isocratic 90%:10% (0 to 1 min), linear from 90%:10% to 10% and 90% (1.1–20.9 min), isocratic 10%:90% (21–25 min), and finally, isocratic 90% and 10% (25.1–28 min) of solvent A and B, respectively. The working solvent (10 µL) was injected as a blank sample. The retention time and masses of the detected phytoconstituents were recorded using Peak view 2.2 software (SCIEX, USA) and MS-DIAL 3.70 software for data processing (Tsugawa, Cajka et al. 2015). Databases including ReSpect-negative (1573 records), HDMB, and NIST libraries were used as references for the tentative identification of compounds. The identification was based on comparing their MS/MS patterns with published compounds in the literature.

### 3.8. Molecular Docking Study

The X-ray crystallographic structure of caspase-3 (PDB ID: IGFW); VEGF-1 (PDB ID: 3HNG); and VEGF-2 (PDB ID: 2QU5, 2P2I, 3EWH, and 1YWN) kinase proteins were downloaded. Default “Structure preparation” was employed after the removal of unwanted residues and ligands on MOE 2016.10. By redocking of the protein ligands, the docking setup was validated for predicting the interactions. The protocol suitability was assured by a low-binding energy score (S) and small RMSD value after being protonated and the energy minimized. The validated protocol was used to predict citrulline–receptor interactions [35].

### 3.9. Statistical Analysis

The results of the VEGF, BAX, BCL-2, and caspase-3 relative mRNA expressions followed a normal distribution, as determined by the Kolmogorov–Smirnov and Shapiro–Wilk tests (*p* < 0.05). Therefore, we employed one-way ANOVA parametric tests and Games–Howell as a post-hoc test because of the heterogeneity of the variances. The mean and standard deviation were used to express continuous variables. GraphPad Prism 8.4.2. (San Diego, CA, USA) was used to conduct the statistical analyses. The cutoff threshold for statistical significance was set at a *p*-value of less than 0.05.

## 4. Conclusions

The current study provided the first insights into the phytochemical profiling of watermelon rind and associated cytotoxic activity. We tentatively identified bioactive compounds belonging to different chemical classes, with organic and phenolic acids being the major constituents besides flavonoids, coumarins, and stilbenes. Phytochemical profiling of the watermelon rind revealed that it is a qualitatively rich source of natural phenolic compounds. Moreover, the rind aqueous extract showed anticancer activity in seven cancer cell lines, as evidenced by its inhibitory effect on cell migration, triggering apoptosis, driving the accumulation of cells in the S phase, and elevating the activity of caspase-3 and the BAX/BCL-2 ratio in HCT116 and Hep2 cells. On the other hand, the rind extracts did not show any antibacterial or antifungal activity. Thus, the complete phytochemical and cytotoxic investigation of the *Citrullus lanatus* rind extract identifies its potential potency as an anticancer agent and may be beneficial as a nutraceutical or food supplement.

## Figures and Tables

**Table 2:** Docked conformations of citrulline on caspase-3 and VEGF kinase proteins.

Proteins	Energy Score (kcal/mol)	No. of Interactions	H-bonding Residues
**VEGF proteins**			
3HNG	−5.09	4	GLU910, CYS912, ASP1040
2QU5	−4.51	4	GLU885, HIS1026
1YWN	−4.33	6	ASP1044, GLU883, HOH123
2P2I	−4.79	6	HIS1026, ASP1046, GLU885
3EWH	−4.19	4	PHE1047, ASP1046
**Caspase-3 protein**			
IGFW	−4.24	8	MET61, GLY122, ARG207

**Table 3:** Primer sequences used for real-time qPCR.

Gene	Accession No.	Primer Sequence	Amplicon(bp)	Melting Temperature (°C)	Annealing Temperature (°C)
VEGF	NM_001025366.3	F-5’-TCCTCACACCATTGAAACCA-3’ R-5’-GATCCTGCCCTGTCTCTCTG-3’	131	56.6 59.3	59.3
Bax	NM_001291430.2	F-5’-ATGGACGGGTCCGGGGAG-3’ R-5’-ATCCAGCCCAACAGCCGC-3’	256	65.6 62.8	62.8
Bcl-2	NM_000657.3	F-5’-AAGCCGGCGACGACTTCT-3’ R-5’-GGTGCCGGTTCAGGTACTCA -3’	258	61.1 61.6	61.1
Casp-3	NM_001354783.2	F-5′-TGGATTATCCTGAGATGGGTTT-3′ R-5′-TTGCTGCATCGACATCTGTA-3′	102	58 55.3	55.3
GAPDH	NM_001357943.2	F-5′-ACCCACTCCTCCACCTTTGA-3′ R-5′-CTGTTGCTGTAGCCAAATTCGT-3′	101	60.8 59.9	59.9

## Data Availability

Not applicable.
